# Optimal fractionation scheme for lymphocyte infiltration in glioblastoma multiforme radiotherapy

**DOI:** 10.3389/fonc.2025.1493436

**Published:** 2025-05-08

**Authors:** Lorea Iturri, Cristéle Gilbert, Julie Espenon, Annaïg Bertho, Sarah Potiron, Marjorie Juchaux, Yolanda Prezado

**Affiliations:** ^1^ Institut Curie, Université Paris Sciences et Lettres (PSL), Centre National pour la recherche scientifique (CNRS) Unité mixte de recherche (UMR3347), Inserm U1021, Signalisation Radiobiologie et Cancer, Orsay, France; ^2^ Université Paris-Saclay, Centre National pour la recherche scientifique (CNRS) Unité mixte de recherche (UMR3347), Inserm U1021, Signalisation Radiobiologie et Cancer, Orsay, France; ^3^ New Approaches in Radiotherapy Lab, Center for Research in Molecular Medicine and Chronic Diseases (CIMUS), Instituto de Investigación Sanitaria de Santiago de Compostela (IDIS), University of Santiago de Compostela, A Coruña, Spain; ^4^ Oportunius Program, Galician Agency of Innovation (GAIN), Xunta de Galicia, A Coruña, Spain

**Keywords:** radiotherapy, glioma, lymphocytes, infiltration, fractionation

## Abstract

**Purpose:**

Radioresistant and immunosuppressive tumors, such as glioblastoma multiforme (GBM), remain a challenge, as current clinical approaches—surgical resection and chemoradiation—do not yet provide effective treatment. *Immunotherapy* (IT) has emerged as a powerful tool in *cancer*; however, phase III clinical trials in GBM have yielded unsuccessful results, likely due to its critical dependence on preexisting antitumor immunity. Given its immunomodulatory potential, radiotherapy (RT) could serve as a tool to induce tumor inflammation and enhance responsiveness to IT. However, the optimal radiation configuration required to achieve the critical level of tumor inflammation for IT success remains elusive. This study assessed the most effective dose fractionation scheme for maximizing immune cell infiltration into tumors.

**Materials and methods:**

Two orthotopic rat glioma models with differing vascularization and immunogenicity were irradiated with three dose fractionation schemes. Tumor immune cell populations were analyzed by flow cytometry.

**Results:**

A single high dose (25 Gy) or extreme hypofractionation is required to elicit a significant immune infiltration in tumors.

**Conclusions:**

Using RT as an immune primer in GBM would require very high and toxic doses with conventional RT methods. While 25 Gy is used in conventional stereotactic radiosurgery, such a high dose is typically limited to small brain volumes. Novel approaches, such as FLASH-RT or minibeam RT, offer alternatives to mitigate toxicity while achieving the required doses.

## 1 Introduction

Glioblastoma multiforme (GBM) remains a challenging condition. The current standard of care—surgery followed by radiation therapy (RT) and chemotherapy with temozolomide (TMZ) (1)—only modestly improves patient survival, and survivors often experience permanent deficits due to normal tissue sequelae. Immunotherapy (IT) has the potential to revolutionize oncology (2), with adoptive cell transfer and checkpoint blockade being the primary strategies in clinical practice. However, its clinical success in solid tumors has often been limited due to various barriers (3), including the irregular stroma and vasculature of these tumors (4), immune-suppressive cytokines and suppressor cells, and T-cell exhaustion. Randomized phase III clinical trials in GBM patients using the immune checkpoint inhibitor (nivolumab) showed no survival improvements (5). Similarly, limited antitumor response was observed using CAR-T therapy in three different clinical trials (6–8).

Increasing evidence over the past several years has highlighted the immunomodulatory role of RT (9). RT can exert either immunosuppressive or immunostimulatory effects on irradiated tumors, depending on the immune context of cancer, total dose, dose per fraction, dose delivery method, and treatment duration. Conventional fractionation schemes (2 Gy per fraction over several weeks) are generally considered immunosuppressive, whereas hypofractionation schemes tend to promote immunostimulation (9). Although not widely used, interest in hypofractionation for GBM treatment is growing (10). The American Society for Radiation Oncology (ASTRO) guideline on radiation therapy for glioblastoma supports its use in elderly patients or those with poor performance status, based on numerous prospective randomized trials (11). Hypofractionation has also been explored for newly diagnosed GBM (either preoperative or postoperative) or recurrent GBM.

Moreover, the success of ICI treatment is well-established to depend on preexisting T-cell infiltration of the tumor (12).

To advance effective radioimmunotherapy combinations for GBM treatment, we conducted an *in vivo* study using two rat glioma models to determine the most favorable temporal fractionation schemes for eliciting significant tumoral T-cell infiltration.

## 2 Materials and methods

### 2.1 Ethical statement

All animal experiments complied with institutional animal welfare and ethical guidelines and were approved by the Ministry of Research (Permit No. APAFIS #36372-2022040609163783 v1). Animals were housed at the Institut Curie animal facility accredited by the French Ministry of Agriculture for rodent experimentation. Cages were enriched with cardboard tunnels.

### 2.2 Tumor inoculation

The RG2-[D74] (CRL-2433™, RRID: CVCL_3581; ATCC^®^, Gaithersburg, MD, USA) and F98 (ATCC-2397TM; ATCC^®^) glioma cell lines, transfected with the luciferase gene and green fluorescent protein (GFP) reporter genes (RG2-Luc-GFP and F98-Luc-green fluorescent protein (GFP)), were used. A total of 50,000 RG2-Luc cells and 10,000 F98-Luc cells were suspended in 5 µL of DMEM and injected intracranially into 6-week-old wild-type Fischer F344 rats (Janvier Labs, Le Genest-Saint-Isle, France) using a Hamilton syringe. The injections were performed through a burr hole in the right caudate nucleus at the following coordinates relatives to bregma: anterior–posterior: − 1 mm; median–lateral: + 4 mm; dorsal–ventral: − 5.5 mm from the skull. The presence of a tumor was confirmed by bioluminescence imaging (BLI) before irradiation. d-Luciferin at a concentration of 150 mg/kg was injected intraperitoneally, and bioluminescence was measured 25 min later (at peak of bioluminescence) using the IVIS spectrum (Perkin Elmer, Houten, The Netherlands). Only rats displaying a BLI signal significantly exceeding the background level on the day before irradiation were enrolled in the study. Group randomization was performed based on BLI signals to ensure comparable average signal intensity across groups.

The clinical status of the animals was monitored five times per week throughout the experiment. Rats displaying classic neurological symptoms associated with tumor progression or experiencing significant weight loss were humanely euthanized using CO_2_ asphyxiation.

### 2.3 Irradiations and dose prescription

Unilateral X-ray conventional irradiation was administered using a small animal irradiator, as previously described (13). Rats were anesthetized with isoflurane (2.5% in air) during irradiation, following prior studies (13). Immobilization was achieved using the Xstrahl immobilized bed (https://xstrahl.com/sarrp/) for head and cranial irradiation. Tumor positioning was guided by the cone-beam computed tomography system of the SARRP machine. Irradiation was performed 14 days after tumor inoculation. Based on our previous studies using magnetic resonance imaging (MRI), tumors at this timepoint are at an advanced stage and voluminous (14), occupying a large portion of the right hemisphere. The aim was to irradiate advanced tumors to better mimic clinical conditions, as GBM is typically diagnosed at an advanced stage). Large beams (1.2 cm^2^) were used to ensure full tumor volume irradiation, with two opposing entry ports. The Muriplan treatment planning system (Xstrahl) was utilized to deliver a homogenous dose to the planned treatment volume. A voltage of 220 kV and a current of 13 mA were applied, with inherent and additional filtrations of 0.8 and 0.15 mm of beryllium and copper, respectively. This resulted in an energy spectrum with an effective energy of 69 keV (13).

Three different temporal schemes were used: one, two, and three fractions. For single-fraction irradiation, the therapeutic dose for our glioma models (25 Gy) (15) was prescribed. To achieve the same biological equivalent dose (BED), doses of 16.5 and 12 Gy were administered in two and three fractions, respectively, at 48-h intervals. The BED was estimated using the linear-quadratic model with an α/β ratio of 10, as in previous studies (16, 17). Radiochromic films were placed on the skin for quality assurance. Each cell line (RG2-Luc and F98-Luc) includes four groups: nonirradiated controls and irradiation with one, two, and three fractions. Control animals were killed on the day of the first irradiation, as in previous studies, to prevent loss of animals (mean survival time in this model is 18 days ± 2 days (18)). Animals were killed 7 days after the first irradiation to analyze tumor immune cell populations using flow cytometry. **Table 1** shows the distribution of animals. The number of animals was lower in the three-fraction group, as they reached the endpoint before day 7 after irradiation, suggesting low treatment efficacy for this scheme.

**Table 1:** Group distribution.

Groups	RG2 model	F98 model
Controls	*N* = 11	*N* = 5
3 × 12.5 Gy	*N* = 4	*N* = 5
2 × 16.5 Gy	*N* = 5	*N* = 7
1 × 25 Gy	*N* = 9	*N* = 6

An additional group of F98-bearing mice (*N* = 3) was irradiated with a single 12-Gy fraction to explore the potential detrimental effects of multiple irradiation fractions or a shorter interval between the final irradiation and the killing.

### 2.4 Analysis of tumor immune cell populations by flow cytometry

Tumors were harvested from rats, weighed (see **Supplementary Figure S1**), and immediately processed enzymatically and mechanically. They were incubated in a digestive solution containing Dulbecco’s phosphate-buffered saline (D-PBS, Gibco, USA), 1 mg/mL Collagenase D (Roche, UK), 0.1 mg/mL DNAse I (Sigma-Aldrich, USA), and 3% fetal calf serum (FCS) for 40 min at 37°C in a tissue dissociator (gentleMACS, Miltenyi Biotec, France). The resulting single-cell suspension was resuspended in flow cytometry staining (FACS) buffer (D-PBS with 0.5% bovine serum albumin [BSA] and 2 mM ethylenediaminetetraacetic acid [EDTA]), then filtered and centrifuged. Samples were resuspended in Debris Removal Solution (Miltenyi Biotec) following the manufacturer’s instructions. Cells were blocked with purified anti-CD32 (FcγRII) as a blocking agent. They were then incubated with a viability stain and immunolabeled in a buffer containing PBS and 3% FCS. **Supplementary Table S1** lists the antibodies used. Counting beads (CountBright™ Plus Absolute Counting Beads, Thermo Fisher, France) were added before acquisition.

Cell profiles were recorded using a multiparameter flow cytometer (Fortessa LSR, BD Biosciences, USA) and analyzed with FlowJo™ v10.6 software (BD Life Sciences, France). Details of the gating strategy are provided in **Supplementary Figures S2**, **S3**.

Cell counts were extracted from FlowJo and normalized by the tumor weight. To account for dilution from the addition of count beads, the following equation was used: (counted cells) * (50,000/count beads) * (50/50)]/tumor weight. The 50/50 factor represents the portion of the tumor used in the protocol. At least 50 µL was used, but if the tumor volume was too high, only 50 µL was employed.

### 2.5 Statistical analysis

Statistical analysis was conducted using Brown–Forsythe and Welch ANOVA, with multiple comparisons performed via an unpaired *t*-test with Welch’s correction. These analyses were carried out using GraphPad Prism 10 (GraphPad Software, CA, USA, Boston, USA). Data from flow cytometry of immune cell populations in tumor samples are expressed as mean ± standard error of the mean.

## 3 Results

**Figure 1:**
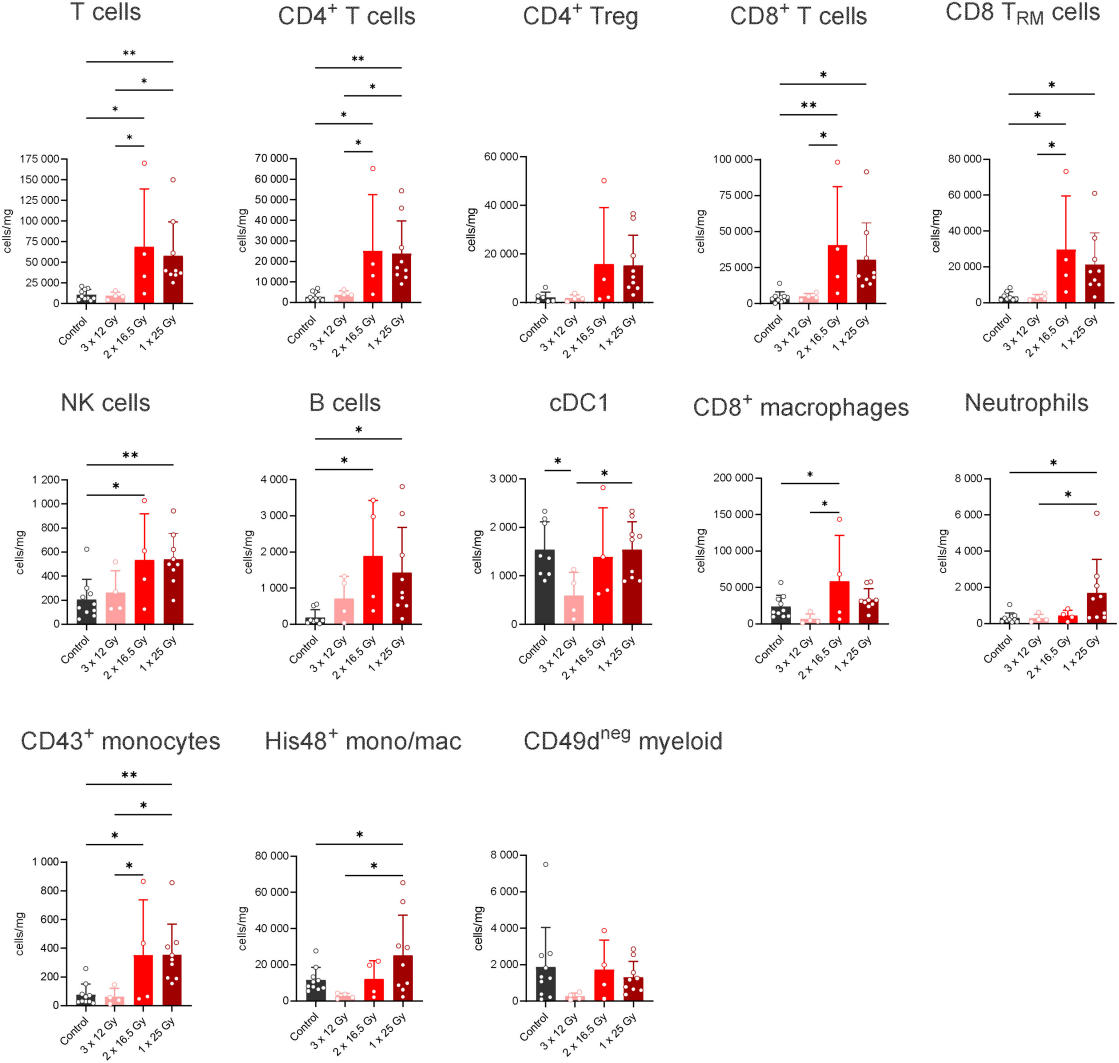
Flow cytometry analysis of immune cells in glioblastoma (RG2 model) across different temporal schemes. See **Supplementary Figure S1** for the gating strategy. * p< 0.05, ** p<0.005.

**Figure 2:**
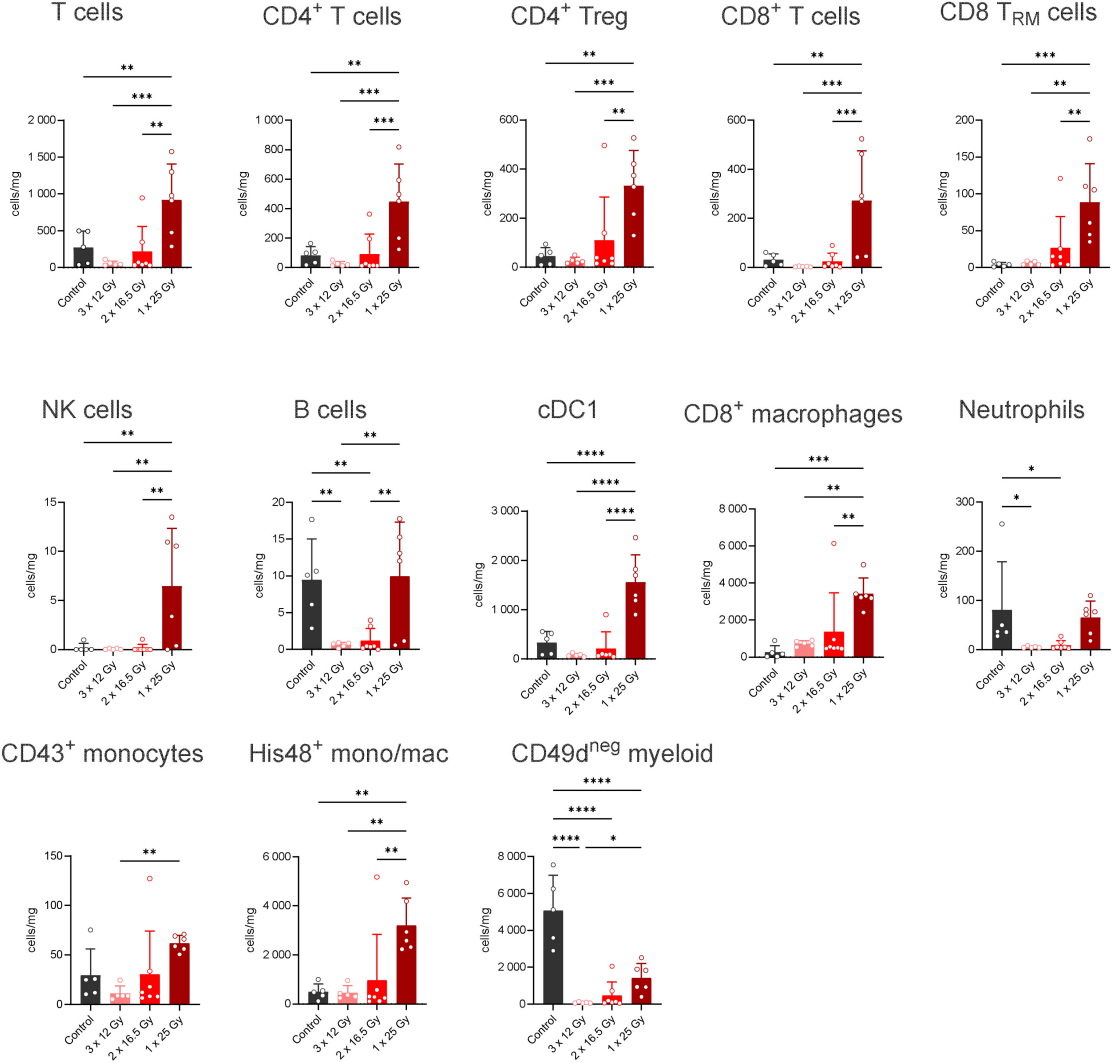
Flow cytometry analysis of immune cells in glioblastoma (F98 model) under different temporal schemes. See **Supplementary Figure S1** for the gating strategy. * p<0.05, ** p<0.005, *** p<0.001 and **** p<0.0001.

**Figures 1**, **2** illustrate the intratumoral immune cell populations in the RG2-Luc and the F98-Luc models, respectively, 7 days after irradiation.

In the RG2-luc model, both one- and two-fraction irradiation schemes result in a significant T-cell infiltration compared to the controls and the three-fraction scheme. A notable infiltration of CD4+ and CD8+ T cells—including CD8 tissue-resident memory T cells (TRM)— as well as natural killer (NK) cells, B cells, and CD43+ monocytes is observed. When two fractions are applied, there is a significant infiltration of CD8+ macrophages compared to the controls and the three-fraction scheme, whereas this is not observed with a single fraction. Conversely, neutrophils show significant infiltration under the one-fraction scheme.

**Figure 3:**
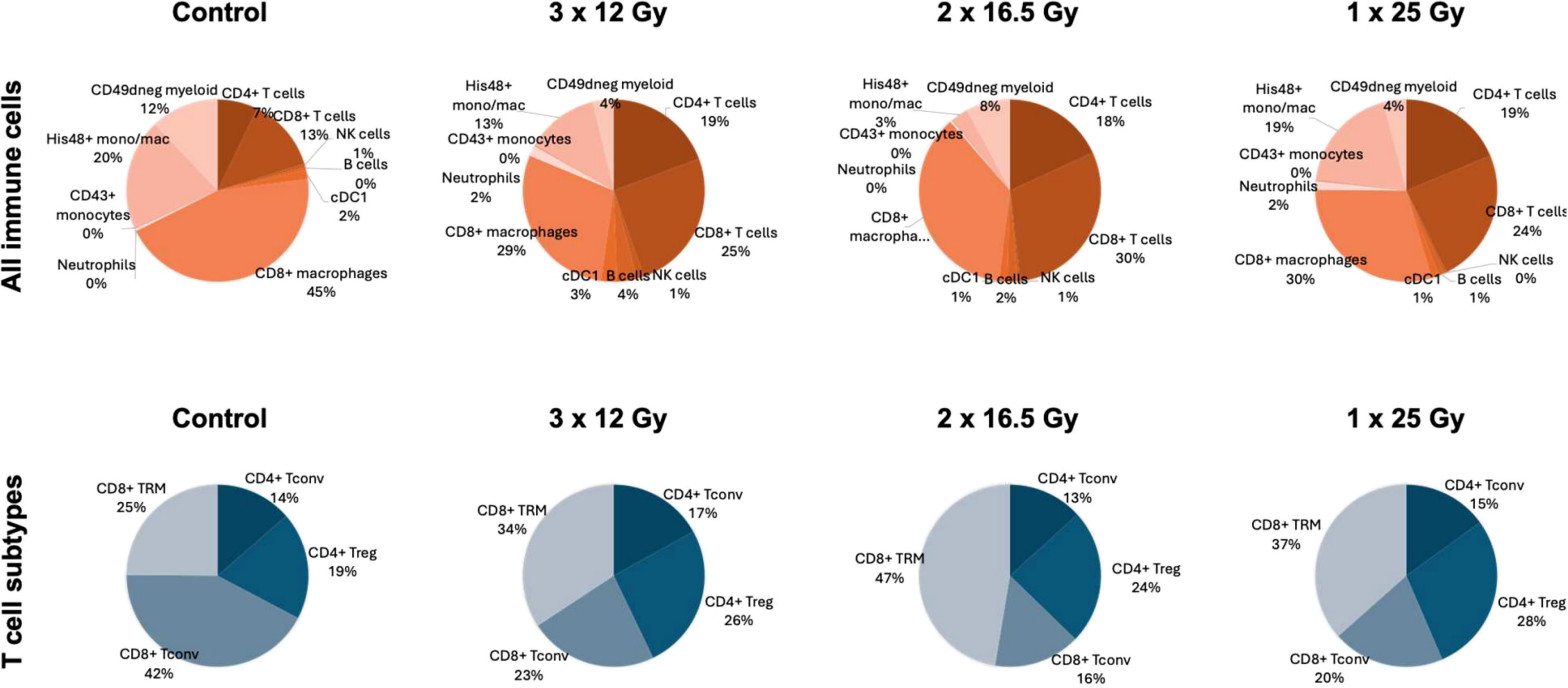
Pie charts depicting the average proportions of immune cells in the RG2 model. Top: Proportions among all CD45-expressing immune cells. Bottom: Proportions among all CD3-expressing T cells. CD4+ T_conv_, CD4+ T cells negative for CD25 (Treg); CD8+ Tconv, CD8+ T cells negative for CD103 (TRM).

**Figure 3** presents the cell density proportions for each cell type and irradiation mode in the RG2 model. Regardless of the fractionation scheme, irradiation reduces the proportion of tumor macrophages and myeloid cells while increasing the proportion of CD8+ and CD4+ T cells. The overall percentage of NK, DC, and B cells remains very low compared to the other evaluated cell types.

In F98-luc tumors, immune infiltration is significantly lower compared with the RG2-Luc tumors, with a 10-fold reduction observed in several cell types). In this model, only a single 25-Gy fraction induces a significant intratumoral infiltration of T cells. Although NK and B cells increased, their levels remained very low. Dendritic cells (cDC1), monocytes, and macrophages were also affected. Irradiation had a strong impact on microglia (CD49d^neg^), an effect not observed in RG2-Luc tumors.

**Figure 4:**
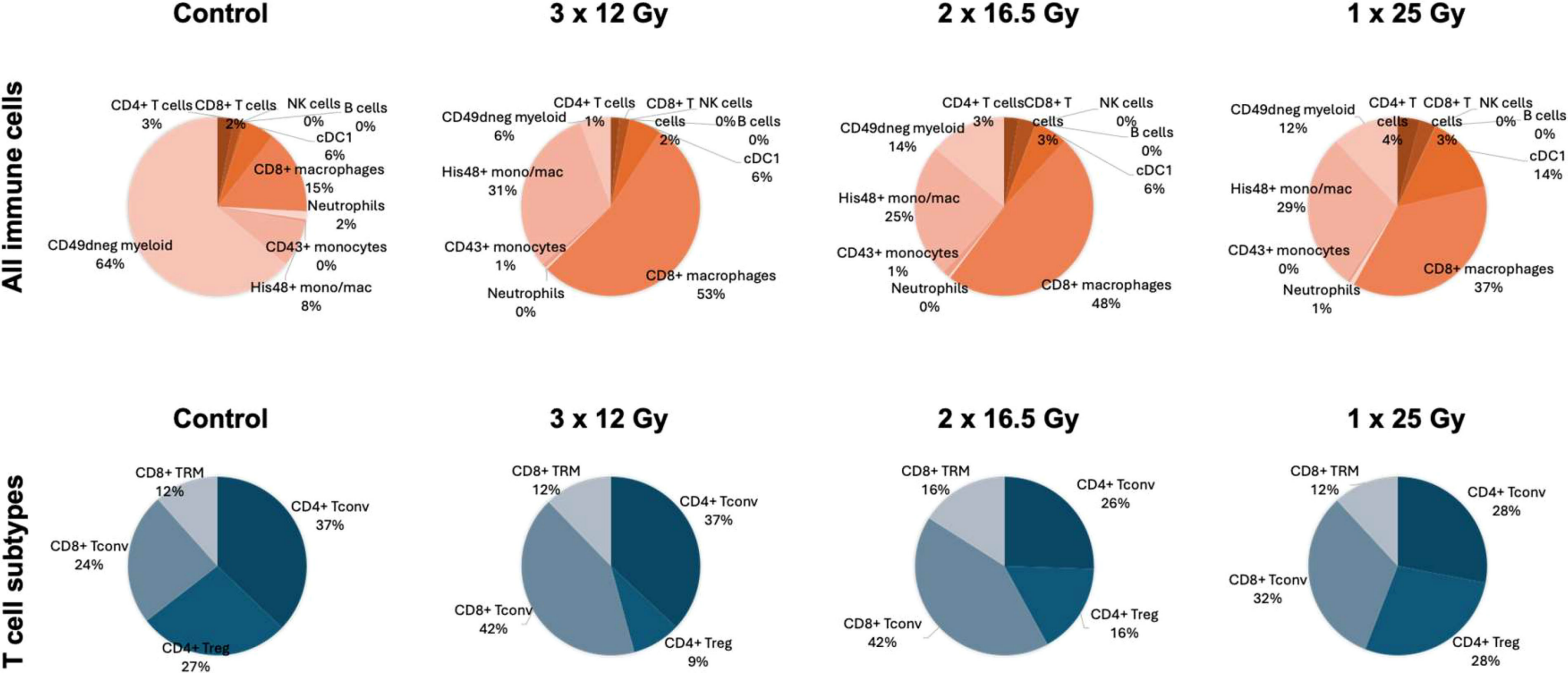
Pie charts showing the average proportions of different immune cells in the F98 model. Top: Proportions among all CD45-expressing immune cells. Bottom: Proportions among all CD3-expressing T cells. CD4+ T_conv_, CD4+ T cells negative for CD25 (Treg); CD8+ Tconv, CD8+ T cells negative for CD103 (TRM).

**Figure 4** presents the proportion of different intratumoral immune cells in the F98 model as a function of the irradiation mode. In the absence of treatment, the most abundant intratumoral cells in the F98 model are microglia-derived CD49neg cells, which are highly immunosuppressive. Unlike the RG2 model, CD8+ macrophages constitute the dominant population. Additionally, the myeloid-to-lymphocyte cell ratio is significantly higher in the F98 model compared to the RG2 one. Irradiation alters the distribution of myeloid cells, markedly reducing the number of CD49neg cells while increasing His48monomac and CD8+ macrophages, with the latter being more pronounced in the 3 × 12 Gy group. Additionally, an increase in CD4+ T cells, CD8+ T cells, and cDC1 is observed in the 25-Gy group. NK and B cells account for less than 1% of the total immune cells analyzed. No significant differences were observed between nonirradiated controls and animals receiving a single 12 Gy fraction (results not shown). A comparison of lymphocyte infiltration (main populations) between controls and animals treated with a single 12-Gy fraction is provided in **Supplementary Figure S5**.

## 4 Discussion

Given its immunomodulatory potential, RT could serve as a tool to induce tumor inflammation and enhance responsiveness to IT. However, the optimal radiation configuration required to achieve the critical level of tumor inflammation for IT success remains elusive.

To advance GBM treatment, we conducted an initial assessment of tumor immune infiltration resulting from hypofractionation schemes in two glioma models. The RG2 model is more vascularized (closer capillaries) (19). F98 glioma models rely on preexisting blood vessels for nutrient supply, whereas RG2 tumors do not alter the length or diameter of major vessels but do induce the formation of new blood vessels within the tumor (20).

F98 is more immunologically excluded and contains a limited amount of T lymphocytes within the tumor (see **Supplementary Figure S4**) (21). To the best of our knowledge, no preclinical evaluation studies have evaluated tumor immune infiltration in glioma-bearing animals (orthotopic models) under different dose fractionation schemes.

Our results indicate that either a single high dose or extreme hypofractionation (2 × 16.5 Gy) is necessary to elicit significant immune infiltration in the tumor. Consistently, we have observed immune infiltration in our previous studies using a single high-dose fraction (18). These findings contradict those of Vanpouille-Box et al. (21), who reported that high doses repress the IFN I pathway and tumor lymphocyte infiltration. The disagreement might be explained by differences in the tumor models used in the two studies (GBM versus mammary carcinoma), as well as the use of orthotopic rather than subcutaneous models. Additionally, this study showed not only the proportion of cells in the tumor but also the density of each individual cell type, allowing for the analysis of population increases independently of changes in neighboring populations.

The infiltration is considerably lower in the F98-GFP-luc model than in the RG2-GFP-luc one. This may be due to reduced tumor vascularization and greater immunosuppression. Notably, the relative increase of Tregs compared to controls and other cell types is higher in the F98-Luc model than in the RG2-Luc one (see **Figures 1**, **3**). Importantly, CD8+ T-cell density, which plays a crucial role in IT success, is very low in the F98-cell line.

Finally, since our results indicate that one or two very high doses may increase lymphocyte infiltration in GBM, an alternative delivery mode should be considered to mitigate the extreme toxicity of high radiation doses to the brain. Minibeam radiation therapy could be a promising and safe option for immunologically cold tumors in patients (18).

This study includes only three types of fractionations and doses. Further experiments varying these parameters, including different tumor stages, are warranted.

## Data Availability

The original contributions presented in the study are included in the article/**Supplementary Material**. Further inquiries can be directed to the corresponding author.
