# Supplementary material for: Optimal fractionation scheme for lymphocyte infiltration in glioblastoma multiforme radiotherapy
Source: Front Oncol. 2025 May 8;15:1493436. doi: 10.3389/fonc.2025.1493436 (PMC12095198; doi:10.3389/fonc.2025.1493436)
Supplement: Supplementary file 1 [file DataSheet1.docx]

Supplemental Materials

***Supplemental Figure S1.*** *Tumor weights of the diffetent groups at the moment of sacrifice. Left: RG2-tumors. No statistically significant difference is observed. Right: F98-tumors. The tumors receiving 3 x 12 Gy presented heavier tumors, coherent with the lack of control observed in that group.*


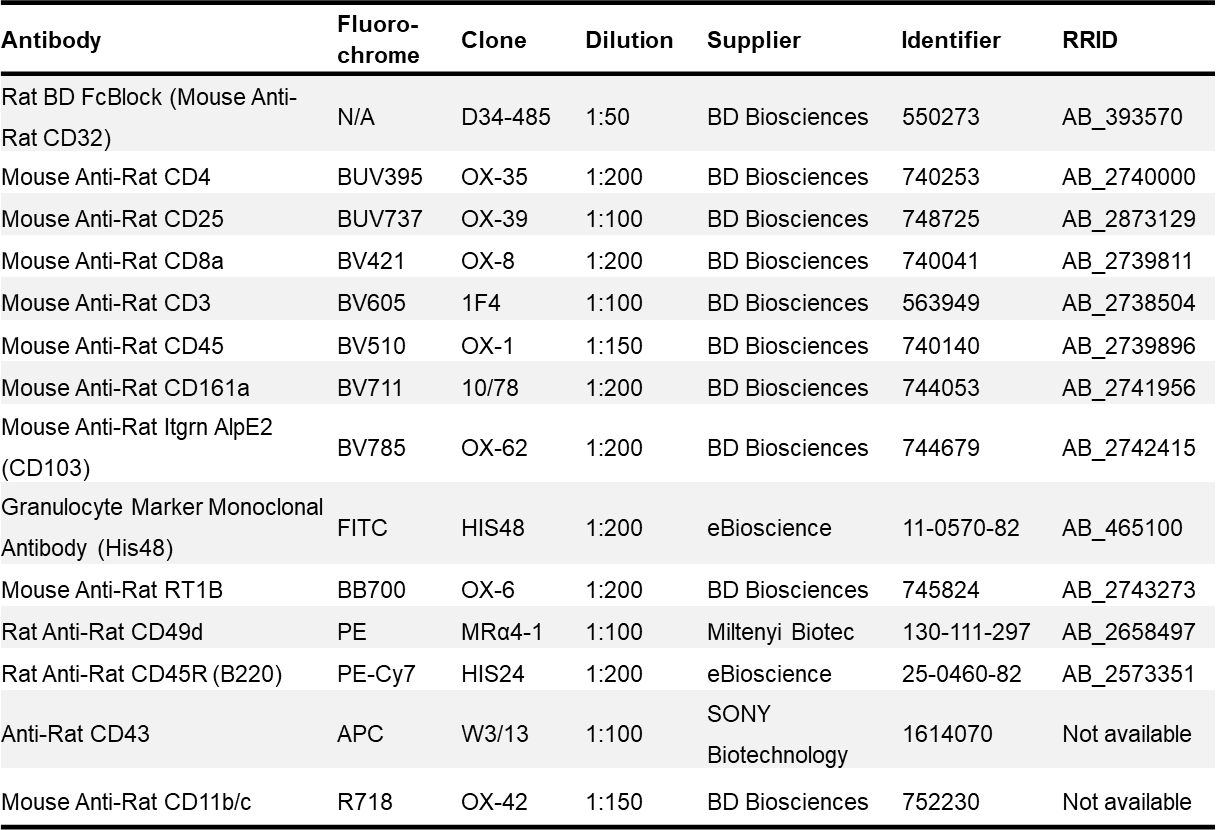


*List of antibodies for flow cytometry*

***Supplementary Table S1****: List of antibodies for flow cytometry on tumoral tissues used in this study.*


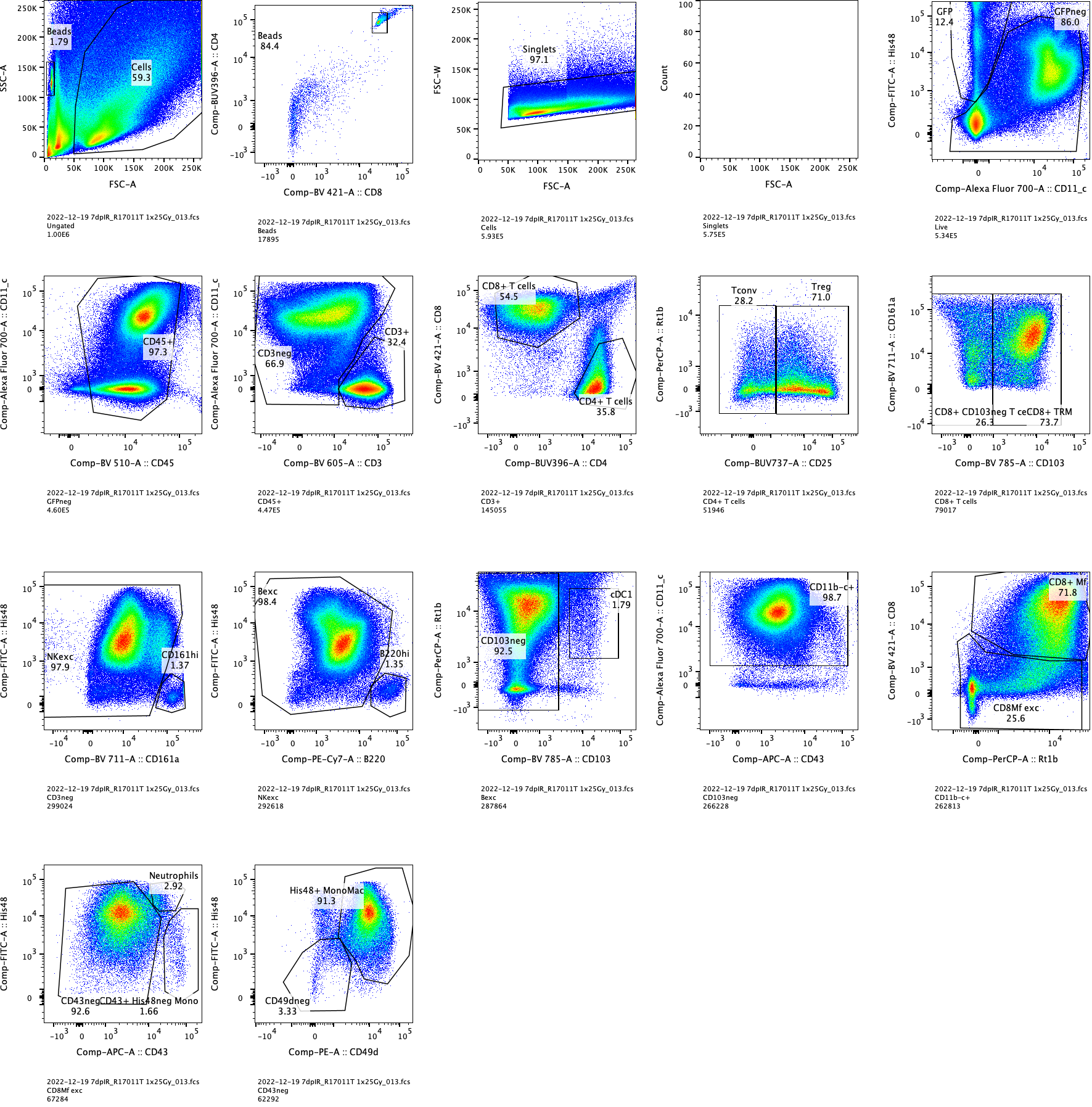


***Supplementary Figure S2:*** *Gating strategy employed for the analysis of intratumoral immune cells. Representive sample of irradiated RG2 glioblastoma 7 days after cranial irradiation.*

*
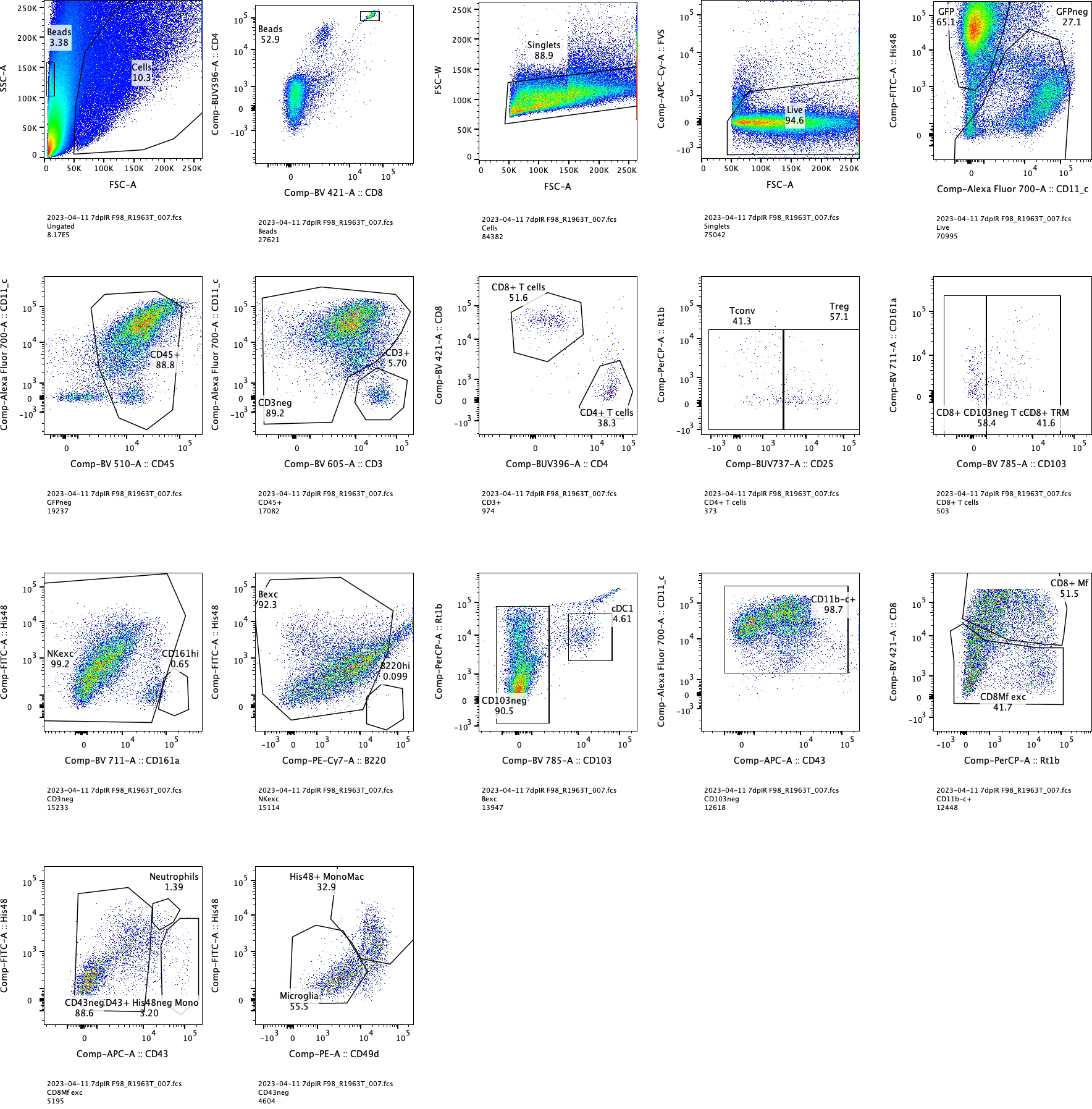
*

***Supplementary Figure S3:*** *Gating strategy employed for the analysis of intratumoral immune cells. Representive sample of irradiated RG2 glioblastoma 7 days after cranial irradiation.*

***Supplemental Figure S4:*** *Comparison of cell densities of different populations between the two tumor types (RG2 and F98) in the the control groups*

***Supplemental Figure S5:*** *Comparison of cell densities of different lymphocytes populations* between non irradiated controls and the animals receiving 1x 12 Gy.
